# Auricular Acupressure for Preventing Postoperative Catheter‐Related Bladder Discomfort in Male Patients Undergoing Spinal Surgery: A Randomized Controlled Trial

**DOI:** 10.1155/nrp/3182987

**Published:** 2026-06-24

**Authors:** Yang Dong, Dingde Long, Bei Fang, Shui-Gen Song, Peng Liao, Tian-yuan Li, Lei Li

**Affiliations:** ^1^ Department of Anesthesiology, The First Affiliated Hospital, Jiangxi Medical College, Nanchang University, Nanchang, 330006, Jiangxi, China, ncu.edu.cn; ^2^ Department of Anesthesiology, People’s Hospital of Jinggangshan, Jinggangshan, 343604, Jiangxi, China; ^3^ Department of Traditional Chinese Medicine, The First Affiliated Hospital, Jiangxi Medical College, Nanchang University, Nanchang, Jiangxi, China, 330006, ncu.edu.cn

**Keywords:** auricular acupressure, catheter-related bladder discomfort, randomized controlled trial

## Abstract

**Aims and objectives:**

This present study evaluated the utility of auricular acupressure (AA) in alleviating catheter‐related bladder discomfort (CRBD).

**Background:**

The efficacy and safety of current treatments for postoperative CRBD in male patients remain suboptimal.

**Design:**

A randomized, placebo‐controlled trial was conducted.

**Methods:**

This trial enrolled male patients postlumbar surgery from 1 July 2024 to 31 August 2024. Participants were allocated to an AA group (Group AA, *n* = 40) or a placebo control group (Group C, *n* = 40). Both groups received 3 days of intervention prior to surgery. Outcomes included the following: Moderate‐to‐severe CRBD at extubation; 24‐h postoperative opioid requirement; CRBD severity (none/mild/moderate‐to‐severe) at 1, 6, and 24 h postextubation; postoperative pain; and patient satisfaction. Data were analyzed via SPSS 23 using *t*‐tests, chi‐square tests, and repeated‐measures ANOVA.

**Results:**

In this randomized trial of 80 male spinal surgery patients, AA significantly reduced moderate‐to‐severe CRBD incidence at extubation (T1): 22.5% (9/40) in Group AA vs. 52.5% (21/40) in Group C (RR: 0.263; 95% CI: 0.100–0.691; *p* = 0.006). AA decreased the subjects of 24‐h supplemental opioid requirements (17.5% vs. 37.5%; RR: 2.829; 95% CI: 1.003–7.977; *p* = 0.045) and elevated patient satisfaction (median 5.0 vs. 4.0; *p* = 0.034). No between‐group differences existed in morphine‐equivalent consumption or CRBD severity beyond T1 (*p* > 0.05).

**Conclusions:**

AA is an effective, nonpharmacological intervention that reduces early postoperative CRBD severity and opioid demand while enhancing recovery satisfaction in catheterized males. As a noninvasive, side‐effect‐free intervention, AA warrants integration into enhanced recovery after surgery (ERAS) protocols for catheterized patients.

**Trial Registration:** Chinese Registry of Clinical Trials: ChiCTR2400086089.

**Relevance to Clinical Practice**
• AA is an effective, nonpharmacological intervention that reduces early postoperative CRBD severity.

## 1. Introduction

A significant number of spinal surgery patients require Foley catheters. In line with advancements in comfort‐oriented care, urinary catheterization is increasingly performed under general anesthesia to reduce pain and distress associated with awake insertion [1]. However, catheterization during anesthesia often leads to pronounced burning sensations, urinary urgency, and frequency postsurgery. Patients may also experience irritability, restlessness, and, in severe cases, behavioral disturbances such as attempted catheter removal. These symptoms are collectively termed catheter‐related bladder discomfort (CRBD) [2–4]. Evidence indicates that catheterization under anesthesia substantially increases the risk of CRBD in the postanesthesia care unit (PACU) [5].

CRBD arises from involuntary bladder contractions mediated through several pathways: stimulation of urethral *M* receptors triggering smooth muscle contraction, prostaglandin secretion, and residual anesthetic effects blunting conscious response to discomfort, thereby facilitating CRBD [2, 6–9]. This condition can exacerbate postoperative pain, increase complications, impair recovery quality, and prolong hospitalization [10], underscoring the need for early prevention and management [11, 12].

Although pharmacological agents such as ketamine, tolterodine, dexmedetomidine, and tramadol have been investigated for CRBD prevention [13, 14], incidence remains high (47%–90%) [15–17], and these treatments often cause side effects, including dry mouth, flushing, blurred vision, and sedation [10]. An effective intervention without adverse effects remains elusive [18, 19].

Auricular acupressure (AA) represents a noninvasive, economical, and side‐effect‐free alternative [20]. Rooted in meridian theory, AA stimulates reflex points on the ear that have neurophysiological connections to the autonomic and central nervous systems, promoting the flow of Qi and blood and restoring balance [21, 22]. Previous studies indicate AA can aid sedation, alleviate pain, regulate autonomic function, and reduce anxiety [23–25]. Nonetheless, its efficacy in preventing postoperative CRBD has not been studied.

Given that male gender, larger catheter size, and absence of analgesics increase CRBD risk [26], this study selected male patients undergoing spinal surgery to minimize confounding from urological procedures. We hypothesized that perioperative AA could reduce the incidence and severity of CRBD and aimed to evaluate its effectiveness in this population.

## 2. Methods

### 2.1. Study Design and Settings

This double‐blind, randomized, placebo‐controlled trial was executed at the First Affiliated Hospital of Nanchang University. The Ethics Committee of the First Affiliated Hospital of Nanchang University approved the study protocol (2024 [279]). The trial was registered at https://www.chictr.org.cn prior to participant enrollment (No. ChiCTR2400086089; Principal investigator: L.T.Y; registration date: June 18, 2024). Participants provided written informed consent.

### 2.2. Subjects

We recruited male patients scheduled for spinal surgery, not expected to exceed 4 h. Eligible patients were aged 18–60 years, with a body mass index (BMI) of 18.5–24 kg/m^2^ and an American Society of Anesthesiologists (ASA) physical status ≤ II using 14 or 16 French (Fr) Foley catheters post‐PACU extubation. Exclusions applied to individuals with severe organ dysfunction (e.g., severe liver or renal dysfunction), preoperative dysuria (e.g., myelopathy, prostate hyperplasia, urethral stenosis, and infection), psychiatric history, allergies to medical tapes, postoperative blood pressure below 20% of baseline, prolonged anesthetic recovery exceeding 2 hours, or other conditions deemed unsuitable.

### 2.3. Randomization and Masking

Using a computer‐generated random number table, patients were randomized 1:1 into the placebo‐controlled (Group C) group or the AA (Group AA) group. Random allocations were sealed in sequential opaque envelopes under trial coordinator supervision. When eligible participants were identified, the coordinator, uninvolved in treatment or data evaluation, opened the envelope and readied auricular plasters with vaccaria seeds. To maintain blinding, the acupressure specialist who applied the plasters and instructed the participants was not involved in outcome assessment. Participants in Group C received the same auricular plasters with *Vaccaria* seeds affixed to the same acupoints but were instructed not to apply pressure. All participants were informed that they might receive either active or sham acupressure. Outcome assessors, anesthesiologists, ward nurses, and the statistician were blinded to group allocation. The acupressure specialist was not blinded due to the nature of the intervention. Anesthesiologists, healthcare team members, and the investigator responsible for recruitment, data collection, and follow‐up were also blinded to group assignment. The implementer was not blinded.

### 2.4. Study Interventions

**FIGURE 1:**
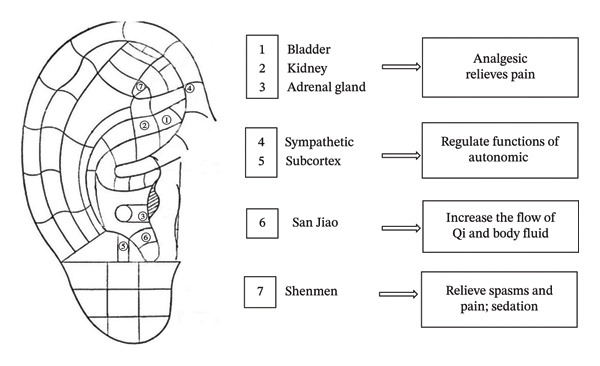
Localization and functions of seven auricular acupoints.

Based on prior pain management research [27, 28], a 3‐day AA application was recommended for this study. The intervention was administered for three consecutive days prior to surgery (i.e., on Day‐2, Day‐1, and on the morning of surgery before anesthesia induction). The AA and C groups underwent true and sham AA, respectively, over the 3‐day preoperative period. The AA protocols were constructed following the Medical Research Council Framework for Developing and Evaluating Complex Intervention [29], reflective of AA theories, CRBD symptomatology, and evidence from reviews, trials, and practice standards [30]. Development and validation details of the AA intervention can be found in a separate paper [30], while true and sham AA protocols are briefly outlined in the following. In the procedure, researchers practiced hand hygiene before AA, cleansing the patient’s ear with a saline‐moistened cotton pad. Seventy‐five percent alcohol swabs disinfected reflex points preoperatively. True AA was administered on the 3 days leading up to surgery, focusing on seven acupoints associated with CRBD relief: “Bladder,” “Kidney,” “San Jiao,” “Shenmen,” “Sympathetic,” “Adrenal gland,” and “Subcortex” (Figure 1). An acupoint detector located these points, with *Vaccaria* seeds affixed using hypoallergenic tape. Participants pressed taped seeds thrice daily for 6 min per session, applying pressure to seeds on both ears. To create a sham control while maintaining consistency in acupoint selection, Group C received the same auricular plasters affixed to the same acupoints but did not perform manual pressure. This design ensured that any difference between groups could be attributed to the mechanical stimulation (pressure) rather than the acupoint selection itself. Intervention was performed by Y.D, trained in AA, and overseen by a traditional medicine expert. Training involved acupoint selection and taping auricular plasters with *Vaccaria* seeds. Intervention adherence was monitored daily by acupressure specialists and researchers.

### 2.5. Study Procedures

Standard monitoring comprised ECG, SpO_2_, IBP, ETCO_2_, BIS, nasopharyngeal temp, and urine output. Standard anesthesia involved sufentanil 0.5 μg/kg and propofol 2 mg/kg for consciousness, followed by cisatracurium 0.2 mg/kg and tracheal intubation, with maintenance via propofol and remifentanil infusions, and cisatracurium for relaxation. Anesthesia depth aimed for BIS between 40 and 60. Fluid infusion was based on routine practices. Intraoperative vasoactive drugs maintained HR and BP within ±20% baseline, followed by flurbiprofen and toltestrone 30 min presurgery completion. A PCIA device was attached postsurgery for sufentanil, toltestrone, and flurbiprofen infusion, delivering 2 mL/h and per‐demand, with a 15‐min lockout. Postsurgery, plasters were removed, patients were transferred to the PACU, and extubated after satisfactory recovery. The primary outcome (moderate‐to‐severe CRBD) was assessed at extubation (T1), which occurred approximately 1 hour after surgery completion. Monitoring continued for SpO_2_, HR, and BP in PACU for 1 h or until a modified Aldrete score of ≥ 9 was attained. In PACU, sufentanil (0.1 μg/kg) was administered for a visual analog scale (VAS) ≥ 4; tramadol (1 mg/kg) treated moderate‐to‐severe CRBD [13, 31]. For simultaneous complaints, either tramadol or sufentanil was administered per dominant complaint, reassessing post‐10‐min administration. Protocol continued postgeneral ward transfer, with BP and pulse oxygen saturation checks until discharge.

### 2.6. Data Collection and Measurements

Participant observation in the PACU or ward was conducted by an anesthesiologist blind to interventions. Baseline data covered patient details (age, weight, height, and ASA physical status). Intraoperatively, anesthesia medications, fluid infusion, urine output, and anesthesia/surgery durations were tracked. Before initiating the trial, the investigator educated all patients on the symptoms of CRBD (urgency and suprapubic discomfort). Subsequently, patients were provided with instructions on how to follow the study protocol and the criteria for evaluating CRBD. CRBD severity was graded as follows: none (no symptoms even upon direct questioning), mild (symptoms reported only upon questioning), moderate (symptoms reported voluntarily without questioning and without behavioral responses), or severe (symptoms reported voluntarily with behavioral responses such as limb flailing, strong vocal response, or attempted catheter removal). This scale is cited from validated literature [13, 32]. Postoperative pain was assessed by a VAS (between 0 and 10, where 0 means no pain and 10 means the worst imaginable pain). Analgesic doses were standardized to IV morphine using equianalgesic conversion (10 mg morphine = 10 μg sufentanil = 100 mg tramadol = 30 mg ketorolac) [33]. Patient satisfaction was gauged 6 h postsurgery using a modified Global Perceived Effect scale (GPES) (1 = very dissatisfied to 7 = very satisfied) [34]. Valid VAS and GPES outcomes were ensured by preoperative rating instructions. Satisfaction analysis subdivided grades (1–3 = dissatisfied; 4 = neutral; 5–7 = satisfied). The primary outcome was the incidence of moderate‐to‐severe CRBD at extubation (T1). Secondary outcomes included the following: CRBD severity at T1 (0 h), T2 (1 h), T3 (6 h), and T4 (24 h); 24‐h supplemental opioid requirement; 24‐h morphine‐equivalent consumption; postoperative pain (VAS); Ramsay sedation score; and patient satisfaction (GPES at 6 h).

### 2.7. Statistical Analysis

#### 2.7.1. Sample Size Estimation

The sample size was calculated based on a previous study [35], with approximately 66% of patients reporting CRBD levels above a moderate grade postoperatively. According to our preliminary trial, the incidence of moderate‐to‐severe CRBD in patients at 0 h after spinal surgery under AA was about 25%. We anticipated that the incidence of moderate‐to‐severe CRBD would be reduced from 66% to 30% in the AA group. A total of 72 subjects were required to provide 80% power at an alpha value of 0.05. Considering a dropout rate of approximately 10%, 80 subjects were planned to be enrolled in this study.

#### 2.7.2. Outcome Analysis

Intention‐to‐treat population analysis was conducted, with per‐protocol following protocol deviations exclusion. Data were presented as mean ± SD, median (interquartile range), number (proportion), relative risk (RR), and 95% confidence interval (95% CI). Categorical variables were assessed with chi‐square, continuity correction, or Fisher’s exact tests. Normally distributed data were analyzed by *t*‐tests and non‐normal by Mann–Whitney U. Primary outcome contrasted with a *χ* ^2^ test, expressing intergroup differences in RR and 95% CI. Analogous per‐protocol analysis was performed. Secondary outcomes were assessed across time points using *χ* ^2^, continuity correction, or Fisher’s exact tests. Opioid use, satisfaction, and Ramsay scores—non‐normal/ordinal variables—were analyzed with Mann–Whitney *U*‐tests. *p* < 0.05 defined significance. Statistical work employed SPSS Version 23 (SPSS Inc.).

## 3. Results

**FIGURE 2:**
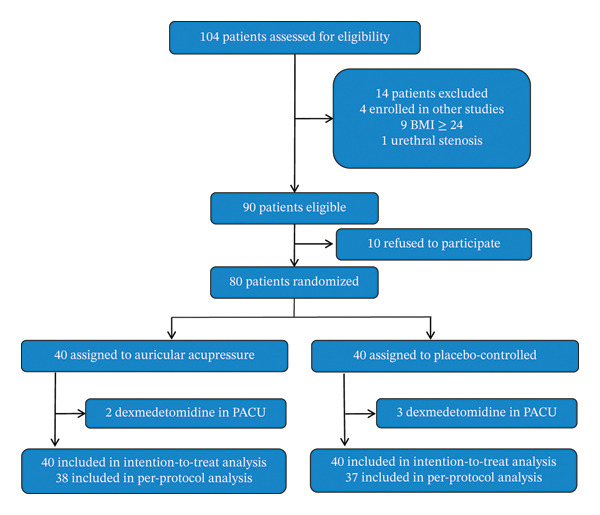
Trial diagram. BMI: body mass index; PACU: postanesthesia care unit.

Between July 1 and August 31, 2024, 104 patients were evaluated for eligibility. Out of 90 eligible participants, 80 were randomized to either placebo‐controlled (*n* = 40) or AA (*n* = 40) groups. Five protocol deviations occurred (five received dexmedetomidine during PACU for agitation not related to CRBD), thus 80 participants were included in the intention‐to‐treat analysis and 75 in the per‐protocol analysis (Figure 2). The inclusion of these five patients in the ITT analysis follows the principle of preserving randomization and avoiding bias from postrandomization exclusions.

Both groups maintained comparable preoperative baseline characteristics (Table 1), while intraoperative metrics—extubation duration, surgery lengths, anesthesia medications, fluid balance, and blood loss—showed no variance (Table 1). Moderate‐to‐severe CRBD at T1 was notably reduced in Group AA (22.5% [9/40]) versus Group C (52.5% [21/40]; RR: 0.263; 95% CI: 0.100–0.691; *p* = 0.006). Per‐protocol results mirrored: 18.4% (7/38) in Group AA versus 48.6% (18/37) in Group C (RR: 0.283; 95% CI: 0.084–0.677; *p* = 0.005; Table 2). Supplemental analgesic need within 24 h was lower in Group AA (17.5% [7/40]) compared to Group C (37.5% [15/40]; RR: 2.829; 95% CI: 1.003–7.977; *p* = 0.045). Morphine‐equivalent use within 24 h showed no disparity (Table 3). No AA‐related adverse events were observed in either group during the study period.

**TABLE 1:** Preoperative baseline and intraoperative characteristics.

	**Group AA (*n* = 40)**	**Group C (*n* = 40)**	**F/*Z***	**p** **value**

Age (years)	49 (37.25–55.00)	48 (34.25–53.00)	−0.83	0.410
Height (cm)	168.73 ± 5.65	168.58 ± 6.28	0.69	0.910
Weight (kg)	64.78 ± 5.26	63.10 ± 7.21	2.32	0.240
Body mass index (kg/m^2^)	23.39 (22.47–23.53)	23.10 (20.54–23.45)	−1.81	0.070
ASA classification (*n*)				
I/II	21/19	16/24	1.26	0.260
Urinary catheter size (Fr) (*n*)				
14/16	38/2	37/3	0.21	0.640
Baseline VAS of pain	2 (2–3)	2 (2–3)	−0.81	0.420
Duration of surgery (min)	145.25 ± 37.85	125.90 ± 39.48	0.66	0.080
Extubation time (min)	22.5 (15–35)	20.0 (15–30)	−0.78	0.430
Intraoperative rocuronium requirement (mg)	45 (35–45)	45 (45–45)	−0.84	0.400
Intraoperative sufentanil requirement (μg)	30 (25–35)	30 (25–35)	−1.58	0.110
Intraoperative remifentanil requirement (mg)	0.78 (0.71–0.85)	0.75 (0.70–0.80)	−1.79	0.070
Intraoperative propofol requirement (mg)	1000 (970–1200)	1000 (972.5–1200)	−0.39	0.640
Urine output (mL)	400 (262.5–500)	300 (262.5–475)	−0.92	0.360
Blood loss (mL)	125 (57.5–200)	90 (50–200)	−1.26	0.210
Total fluids (mL)	1433.75 ± 470.46	1332.50 ± 358.34	3.67	0.280

*Note:* Data are expressed as mean ± SD, *M* (IQR), or numbers (%); Group C, the placebo‐controlled; Group AA, the auricular acupressure.

**TABLE 2:** Incidence and severity of catheter‐related bladder discomfort.

Time group	T1		T2		T3		T4	
	AA	C	AA	C	AA	C	AA	C
Incidence (%)	21 (52.5)	34 (85.0)	20 (50.0)	22 (55.0)	11 (27.5)	16 (40.0)	4 (10.0)	6 (15.0)
*p* value	0.002		0.654		0.237		0.499	
Relative risk (95% CI)	0.195 (0.067–0.567)		0.898 (0.340–1.970)		0.569 (0.222–1.455)		0.630 (0.163–2.427)	
Severity								
Mild	12 (30.0)	13 (32.5)	14 (28.0)	13 (32.5)	8 (20.0)	11 (22.0)	4 (10.0)	6 (15.0)
Moderate to severe	9 (22.5)	21 (52.5)	6 (15.0)	10 (25.0)	3 (7.5)	5 (12.5)	0 (0.0)	0 (0.0)
*p* value	0.003		0.689		0.485		1.000	

*Note:* Data are expressed as numbers (%). Group C, the placebo‐controlled; Group AA, the auricular acupressure; T1, 0‐h extubation of the tracheal tube; T2, 1‐h extubation of the tracheal tube; T3, 6‐h extubation of the tracheal tube; T4, 24‐h extubation of the tracheal tube.

**TABLE 3:** Efficacy outcomes.

	**Group AA (*n* = 40)**	**Group C (*n* = 40)**	**Estimated effect (95% CI)**	**p** v**alue**

Primary outcome				
Moderate‐to‐severe CRBD within T1 (ITT analysis)	9 (22.5)	21 (52.5)	0.263 (0.100–0.691)	0.006
Moderate‐to‐severe CRBD within T1 (PP analysis)	7 (18.4) (*n* = 38)	18 (48.6) (*n* = 37)	0.238 (0.084–0.677)	0.005
Secondary outcomes				
Postoperative morphine equivalent within 24 h (mg)	48 (48–48)	48 (48–66.75)	—	0.056
Supplemental analgesics within 24 h	7 (17.5)	15 (37.5)	2.829 (1.003–7.977)	0.045

*Note:* Data are *n* (%) or median (interquartile range). Group C, the placebo‐controlled; Group AA, the auricular acupressure; T1, 0‐h extubation of the tracheal tube.

Abbreviations: CI, confidence interval; ITT, intention to treat; PP, per‐protocol; RR, relative risk.

CRBD incidence at T1 was significantly reduced in Group AA versus Group C (0 h: 21 [52.5%] vs. 34 [85.0%], *p* = 0.002; RR = 0.195; 95% CI: 0.067–0.567). No significant incidence differences were observed at T2, T3, and T4 for CRBD. Moderate‐to‐severe CRBD incidence showed no significant intergroup difference at T2, T3, and T4 (T2: 25% vs. 15%, *p* = 0.689; T3: 12.5% vs. 7.5%, *p* = 0.485; T4:0% vs. 0%, *p* = 1.000) (Table 3).

Significant differences appeared in VAS and Ramsay scores between Group C and Group AA at T1 (*p* < 0.05); thereafter, VAS and Ramsay scores within T2, T3, and T4 showed no significant difference (*p* > 0.05) (Tables 4 and 5).

**TABLE 4:** Comparison of pain intensity.

	**Visual analog scale**			
	**Group AA (*n* = 40)**	**Group C (*n* = 40)**	**Z**	**p** v**alue**

Time				
T1	2.0 (1.0–3.0)	3.0 (2.0–4.0)	−2.959	0.003
T2	2.0 (1.0–2.0)	2.0 (1.3–3.0)	−2.300	0.021
T3	1.0 (0.3–2.0)	2.0 (1.0–3.0)	−2.743	0.006
T4	1.0 (1.0–1.0)	1.0 (1.0–1.8)	−1.825	0.068

*Note:* Data are expressed as median (interquartile range). Group C, the placebo‐controlled; Group AA, the auricular acupressure; T1, 0‐h extubation of the tracheal tube; T2, 1‐h extubation of the tracheal tube; T3, 6‐h extubation of the tracheal tube; T4, 24‐h extubation of the tracheal tube.

**TABLE 5:** Comparison of Ramsay scores.

	**Ramsay scores**			
	**Group AA (*n* = 40)**	**Group C (*n* = 40)**	**Z**	**p** **value**

Time				
T1	2.0 (2.0–2.0)	2.0 (1.0–2.0)	−2.813	0.005
T2	2.0 (2.0–2.0)	2.0 (2.0–2.0)	−1.140	0.254
T3	2.0 (2.0–2.0)	2.0 (2.0–2.0)	−1.000	0.317
T4	2.0 (2.0–2.0)	2.0 (2.0–2.0)	0	1.000

*Note:* Data are expressed as median (interquartile range). Group C, the placebo‐controlled; Group AA, the auricular acupressure; T1, 0‐h extubation of the tracheal tube; T2, 1‐h extubation of the tracheal tube; T3, 6‐h extubation of the tracheal tube; T4, 24‐h extubation of the tracheal tube.

**FIGURE 3:**
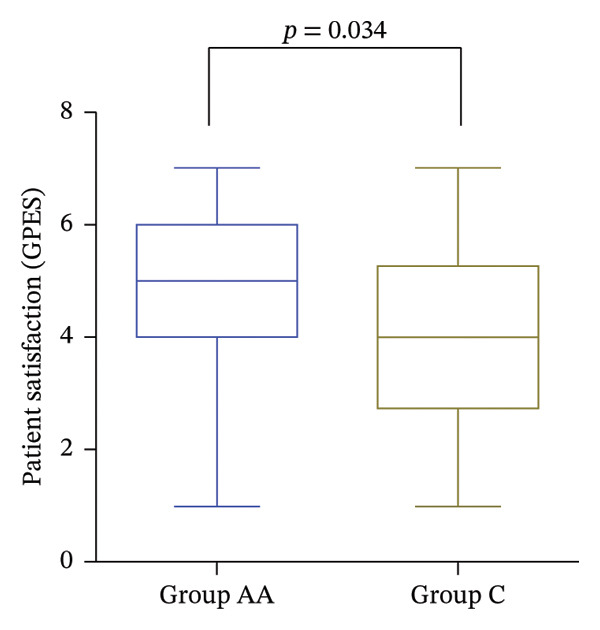
Comparison of patient satisfaction scores between the 2 groups at 6‐h postsurgery. Group C: the placebo‐controlled; Group AA: the auricular acupressure. The horizontal line inside the boxes shows the median values; the upper and lower edges of the box represent the third and first quartiles, respectively. Whiskers above and below the boxes represent 90% and 10%, respectively. Data were analyzed using the Mann–Whitney *U* test. GPES indicates global perceived effects on a 7‐point scale.

Patient satisfaction was markedly higher in Group AA than in Group C (5.0 [2.0–6.0] vs 4.0 [2.0–5.0], *p* = 0.034; Figure 3). Subanalysis by satisfaction grade found more satisfied patients in Group AA versus Group C (60.0% vs. 37.5%) and more dissatisfied patients in Group C versus Group AA (32.5% vs. 12.5%, *p* = 0.028) (Table 6).

**TABLE 6:** Subanalysis according to the grade of patient satisfaction.

	**Group AA (*n* = 40)**	**Group C (*n* = 40)**	**Z**	**p** **value**

Grade of satisfaction			7.124	0.028
Dissatisfied	5 (12.5%)	13 (32.5%)		
Neither	11 (27.5%)	12 (30.0%)		
Satisfied	24 (60.0%)	15 (37.5%)		

*Note:* Data are expressed as numbers (%). Group C, the placebo‐controlled; Group AA, the auricular acupressure. Grade of patient satisfaction: 1 = very dissatisfied, 2 = somewhat dissatisfied, 3 = slightly dissatisfied, 4 = neither satisfied nor dissatisfied, 5 = slightly satisfied, 6 = somewhat satisfied, and 7 = very satisfied. The dissatisfied group included Grades 1, 2, and 3. Neither group included Grade 4. The satisfied group included Grades 5, 6, and 7. Data were compared using the chi‐square test.

## 4. Discussion

Our randomized study identifies AA as a nonpharmacological approach that notably mitigates early postoperative CRBD in male spinal surgery patients. A relative 30% reduction in moderate‐to‐severe CRBD postextubation (T1) (*p* = 0.006), alongside diminished opioid rescue needs and elevated patient satisfaction, demonstrates AA’s dual efficacy in modulating visceral nociception and enhancing perioperative welfare. Importantly, these advantages occur without drug‐related adverse effects, thereby overcoming a significant issue in current pharmacologic treatments for CRBD.

CRBD‐triggered discomfort and abnormal behaviors often evoke agitation postawakening, leading to heightened postoperative complications, such as surgical incision dehiscence, bleeding, circulatory instability, cardiac arrhythmias, extended hospital stays, and increased in‐hospital mortality [10]. CRBD occurs more frequently in males than females [27]; the CRBD incidence in male spinal surgery patients with a 14 or 16 Fr Foley catheter was 85.0%, with moderate‐to‐severe CRBD incidence at T1 reaching 52.5%. Therefore, mitigating postoperative CRBD’s incidence and severity is crucial in perioperative anesthetic management.

### 4.1. Key Findings and Clinical Implications

Reduction of moderate‐to‐severe CRBD**:** Moderate‐to‐severe CRBD incidence at T1 was 52.5% in Group C versus 22.5% in Group AA (*p* = 0.006). This aligns with neurophysiological perspectives that AA activates autonomic pathways (using “Shenmen” and “Sympathetic” points), modulating bladder smooth muscle contraction and nociception [23]. The immediate postextubation phase (T1) signifies peak CRBD severity due to residual anesthesia effects and catheter‐induced urethral stimulation [2, 9]. AA’s effectiveness at this critical juncture underscores its potential for early symptom management.

Reduced opioid requirement**:** AA recipients needed fewer supplemental analgesics within 24 h (17.5% vs. 37.5%, *p* = 0.045). This may be attributed to AA’s facilitation of endogenous opioid release [15, 16], alleviating pain‐related distress and indirectly reducing agitation induced by CRBD [15–18]. The standardized PCA protocol likely accounts for the unchanged morphine‐equivalent consumption.

Enhanced patient satisfaction: Patient satisfaction was significantly higher in the AA group (median 5.0 vs. 4.0, *p* = 0.034), with 60% satisfaction compared to 37.5% in controls. AA’s customer comfort likely arises from its noninvasive character, absence of systemic side effects (such as dry mouth or sedation [10]), and comprehensive symptom relief [21], aligning with modern comfort care principles [1].

### 4.2. Mechanisms of AA in CRBD Management

AA may alleviate CRBD through multifaceted routes: (1) Somatovisceral convergence: Stimulation of ear acupoints (“Bladder” and “San Jiao”) engages auricular branches of the vagus nerve (ABVN), affecting nucleus tractus solitarii and modulating detrusor motor neurons of the sacral spinal cord [23]. (2) Downward inhibition: Acupressure impulses travel to the brain via the meridian line at four times the pain stimulus transmission rate, blocking pain receptor signals and leading to pain relief [36]. “Shenmen” and “Subcortex” point acupressure may enhance endogenous opioid secretion in the periaqueductal gray [37]. (3) Autonomic rebalancing: Sympathetic inhibition through “Sympathetic” point stimulation may lower acetylcholine‐driven detrusor hyperactivity [26], consistent with AA‐identified agitation suppression.

This study faced certain limitations. (1) Sex‐specificity: CRBD susceptibility predominantly in males was examined here, but AA’s efficiency in females—with differing urethral nerve patterns—requires urgent evaluation. (2) Neuromodulation precision: Future trials should employ fMRI to map CNS activity patterns during AA alongside acupoint detectors. (3) Severe CRBD with associated behaviors (limb flailing) may overlap postoperative delirium. Further studies should integrate delirium assessment tools (e.g., CAM‐ICU). (4) Long‐term effects: Intervention duration was limited to 3 days; extended application may prolong benefits past T1. (5) Blinding limitations: Although blinding of outcome assessors and statisticians was maintained, participants in the control group were aware that they did not perform pressure application, which may have introduced performance bias. Future studies should consider using sham acupoints or nonpenetrating sham devices to enhance blinding integrity. (6) Placebo control validity: The control group received plasters on the same acupoints without pressure, which may not represent a fully inert placebo. Although this design helps isolate the effect of pressure stimulation, it does not control for potential nonspecific effects of acupoint application. Future trials should consider using sham acupoints (e.g., nontherapeutic points on the ear) or nonpenetrating sham devices to strengthen the validity of the placebo control. (7) Transient effect: The effect of AA on moderate‐to‐severe CRBD was observed only at extubation (T1) and did not persist at 1, 6, or 24 h postextubation. This transient effect may reflect the acute antinociceptive mechanism of AA, which likely acts through rapid neuromodulation rather than sustained structural changes. While the intervention was applied over 3 days preoperatively, the most pronounced benefit occurred at the time of peak CRBD severity (immediately after extubation), suggesting that AA may be most valuable as an immediate perioperative adjunct rather than as a prolonged prophylactic therapy. Nevertheless, even a transient reduction in moderate‐to‐severe CRBD has clinical significance, as the immediate postextubation period is a critical window during which severe discomfort can lead to agitation, hemodynamic instability, and catheter‐related complications. Future studies should explore whether more frequent or extended stimulation (e.g., continued postoperative stimulation) could prolong the beneficial effect.

## 5. Conclusion

AA effectively diminishes early moderate‐to‐severe CRBD occurrence, curtails opioid use, and boosts patient satisfaction in male spinal surgery patients. As a noninvasive, side‐effect‐free option, AA merits inclusion in enhanced recovery after surgery (ERAS) protocols for catheterized patients. Future research should address study limitations and refine AA protocols for broader surgical demographics.

**Nomenclature**
AA: Auricular acupressure
ASA: American Society of Anesthesiologists
BMI: Body mass index
CRBD: Catheter‐related bladder discomfort
ERAS: Enhanced recovery after surgery
Group AA: The auricular acupressure group
Group C: The placebo control group
PACU: Postanesthesia care unit

## Author Contributions

Tian‐yuan Li: conception and design of the study, data acquisition and interpretation, and manuscript writing and revision. Lei Li: manuscript writing and revision. Bei Fang and Ding‐de Long: study design, data interpretation, and manuscript revision. Yang Dong and Peng Liao: data collection, data analysis, and writing the first draft. Shui‐gen Song: data collection.

## Funding

This work was supported by the Administration of Traditional Chinese Medicine of Jiangxi Province (Grant no. 2021B300).

## Disclosure

All authors approved the final version for publication. All authors agree to be accountable for all aspects of the work and ensure that any questions regarding the accuracy or integrity of any part of the work are properly addressed and resolved.

## Conflicts of Interest

The authors declare no conflicts of interest.

## Data Availability

The datasets generated during and/or analyzed during the current study are available from the corresponding author on reasonable request. All data generated or analyzed during this study are included in this published article.
